# The relationships between depression, inflammation and self-reported disease activity in IBD and their impact on healthcare usage

**DOI:** 10.1186/s12876-025-03691-8

**Published:** 2025-03-06

**Authors:** Natasha Seaton, Vari Wileman, Christine Norton, Joanna Hudson, Valeria Mondelli, Rona Moss-Morris

**Affiliations:** 1 Psychology, Institute of Psychiatry, Psychology and Neuroscience, King’s College London, 5th Floor Bermondsey Wing Guy’s Campus, London Bridge, SE1 9RT London, UK; 2 Nursing Midwifery & Palliative Care, King’s College London, London, UK; 3 Psychological Medicine, Institute of Psychiatry, Psychology and Neuroscience, King’s College London, London, UK

**Keywords:** Inflammatory bowel disease, Crohn’s disease, Ulcerative Colitis, Depression, Self-reported disease activity, Faecal calprotectin, Healthcare usage

## Abstract

**Background:**

Depression is common in people living with Inflammatory Bowel Disease (IBD). Depression rates increase with active disease and are linked to poorer clinical outcomes. Previous studies investigating the relationship between contemporaneous IBD disease activity and depression are often poorly controlled, use small samples and/or rely on self-reported measures of disease activity. Depression and self-reported disease activity (SRDA) are linked to increased healthcare usage, however, objective inflammation is rarely statistically controlled. The primary aim was to understand how self-reported disease activity and inflammation are related to depression. Secondary aims included assessing the relative influence of self-reported disease activity, inflammation and depression on healthcare usage.

**Methods:**

This was a cross-sectional analysis of baseline data collected as part of a randomised controlled trial (trial registration no: ISRCTN71618461) of a digital treatment for symptom self-management in IBD (*n* = 599). Bivariate associations of demographic and clinical variables with depression were conducted to identify relevant covariates. Multiple linear regressions assessed (i) the relationships between depression (Patient Health Questionnaire-9 (PHQ-9)), SRDA (IBD-Control) and intestinal inflammation (faecal calprotectin (FCP)) and (ii) whether these variables explained variance in healthcare usage and economic indicators.

**Results:**

Depression was significantly predicted by SRDA (*β =* -0.82, *p* < 0.001) but not FCP, with the model explaining 37% of the variance in depression (F(2,596) = 175.1, *p* < 0.001). FCP was only weakly associated with SRDA (*r* = -0.16, *p* < 0.001). Depression was independently associated with visits to primary care (*β =* 0.19, *p* < 0.001), IBD secondary care (*β =* 0.13, *p* < 0.001), IBD-related A&E attendance (*β =* 0.10 *p* < 0.05) and the impact of IBD on productivity (*β =* 0.24 *p* < 0.001) in the last 3 months.

**Conclusions:**

Depression was related to SRDA but not FCP. Depression was also associated with healthcare usage even when SRDA and inflammation were statistically controlled. Routinely assessing and treating depression in IBD alongside managing inflammation may improve symptoms for patients and reduce healthcare costs.

**Supplementary Information:**

The online version contains supplementary material available at 10.1186/s12876-025-03691-8.

## Introduction

Inflammatory Bowel Disease (IBD) refers to a group of autoimmune diseases, including Crohn’s Disease and Ulcerative Colitis, where maladaptive immune responses result in gastrointestinal inflammation [1, 2]. IBD manifests as a relapsing and remitting condition, with periods of activity (called ‘flares’) associated with increased symptom report [3]. Symptoms include gastrointestinal symptoms, such as abdominal pain, urgency, diarrhoea and rectal bleeding, but also include systemic issues including depression and fatigue [4]. Depression is prevalent, affecting 25.2% of IBD patients, rising to 38.9% when disease is active [5]. Symptom burden and psychological stress surrounding the unpredictable and uncontrollable nature of IBD are said to explain high depression rates [6], but it has also been argued that depression is an extraintestinal manifestation of IBD, caused by inflammatory activity [7].

Depression and inflammation may be causally linked in IBD through the gut-brain axis, a bidirectional communication system connecting the gastrointestinal system and central nervous system. Gut-brain mechanisms of action have been reviewed extensively elsewhere [8–10]. In brief, intestinal inflammation in IBD leads to release of pro-inflammatory cytokines, including tumour necrosis factor-alpha (TNF-α), interleukin-6 (IL-6), and interleukin-1 beta (IL-1β) [11, 12]. Cytokines can lead to neuroinflammation by permeating the blood-brain barrier, activating microglia to initiate an immune response and contributing to inflammatory depression symptoms [7, 10, 11]. Gut microbiota dysbiosis can also occur, impairing the production of short-chain fatty acids and neurotransmitters, which may impact mood [7–9]. Moreover, inflammation in the gut can stimulate vagal afferent nerves and the hypothalamic-pituitary-adrenal (HPA) axis which are also implicated in mood regulation and depression aetiology [7, 10].

Moreover, depression may exacerbate IBD pathophysiology, including permeability, motility, sensitivity and secretion of the intestine, as well as altering microbiota composition and increasing intestinal inflammation [8]. Clinical studies indicate that depression independently predicts poorer IBD outcomes when controlling for baseline disease activity [13]. Treatments for depression have shown reductions in inflammatory markers like faecal calprotectin (FCP) [14], a non-invasive, highly sensitive marker of inflammation frequently used in IBD management [15].

Despite this, there is limited evidence for contemporaneous relationships between depression and disease activity [16]. In a meta-analysis, disease activity and depression were significantly associated; however, included studies are typically poorly controlled, underpowered, and rely on self-reported disease activity (SRDA) measures [17, 18], such as the Harvey Bradshaw Index [19] or the Crohn’s Disease Activity Index [20] for Crohn’s Disease and the Simple Clinical Colitis Activity Index [21] for Ulcerative Colitis; or the IBD-Control [22, 23] for any IBD subtype. These patient-reported outcome measures (PROMs) are increasingly being used clinically to assess disease activity, support patient-centred care, and inform treatment decisions [24, 25].

Despite these advantages, PROMs often correlate poorly with objective disease indicators of inflammation, such as endoscopic activity, FCP and C-Reactive protein (CRP) [26–29]. Instead, a systematic review has demonstrated that depression, anxiety, perceived stress and pain catastrophising are strongly and significantly associated with self-reported symptoms [6], even when objective disease activity is controlled [30–32]. This may be due to psychological factors altering sensory processing, attention, or pain perception [30, 31]. Depression may increase symptom perception and the reporting of symptoms through psychological, physiological, and neurobiological mechanisms [33]. Depression is related to somatosensory amplification, whereby heightened sensitivity to bodily sensations lowers detection and pain thresholds for sensations [34]. Depression may also cause activation of the HPA axis which elicits physiological sensations as well as heightening symptom awareness [35]. Moreover, negative cognitive interpretations common in depression, such as catastrophizing and selective attention to distressing stimuli, can lead to the magnification of physical sensations [36]. Additionally, neurobiological overlap between brain regions involved in pain perception and mood regulation, such as the anterior cingulate cortex, may exacerbate physical symptom perception [33, 37, 38]. As such, if clinicians rely heavily on PROMs, treatment decision making may be problematic if patients receive biomedical treatments when their self-reported symptoms are driven in part by emotional and psychological factors. Behavioural interventions, either targeting distress, self-management and/or symptoms in remission may be more appropriate [14, 39–43].

Depression is also routinely associated with negative health outcomes, healthcare costs, healthcare usage and absenteeism at work [44–47]. One prospective study found that IBD patients with a psychiatric diagnosis had twice the odds of high IBD-related expenditure, even when disease severity and complexity were controlled [45]. A meta-analysis of prospective studies pooled data from 9192 IBD patients and showed that depression increased the risk of hospitalisation, emergency department attendance and medical escalation [46]. However, studies rarely collect objective inflammation, so it is unclear whether the relationship between depression and negative physical health outcomes is explained by greater levels of objective inflammation or SRDA, or if depression is an independent risk factor for negative health outcomes. Understanding these relationships will enable clinicians to use appropriate, evidence-based gut-brain axis treatments.

### Rationale for the study

The gut-brain axis is highly relevant in the understanding of IBD prognosis and symptomatology. Depression is common in people living with IBD, yet existing research, focussed on the contemporaneous relationships between objective inflammation, depression and SRDA, are limited by inadequately controlled analyses [48]. The primary marker in this study was FCP, chosen for its specificity with IBD disease activity (compared with CRP) as well as its convenience, low cost and non-invasiveness (compared with endoscopy) [49] and strong correlation with endoscopic disease activity [50]. Moreover, it is a good potential candidate to explore associations with depression as IBD inflammation is related to systemic immune activation, which has been shown to influence neurological and psychological processes, contributing to depressive symptoms. Thus, FCP serves as an objective measure of mucosal inflammation while enabling investigation into relationships between inflammation and depression. Despite associations with endoscopy, FCP is limited in detecting small bowel inflammation, common in Crohn’s Disease [51–53]. Therefore, to address previous issues with inadequately controlled analyses, this study included disease subtype as an effect modifier of FCP, as well as other relevant confounders. When assessing contributors to healthcare usage, inflammation is rarely collected. This prevents the assessment of the relative salience of SRDA and objective inflammation in treatment and healthcare usage.

### Research aims

1. Explore the relationships between depression, objective inflammation and SRDA in people living with IBD.
  - Assess how much variance in depression is explained by SRDA and objective inflammation, when controlling for relevant disease and demographic factors.
2. Explore whether depression, SRDA and objective inflammation predict health service use and their relative influence.

## Methods

This study is a cross-sectional analysis of baseline data collected within a UK National Institute of Health Research (NIHR; RP-PG-0216-20001) two-arm randomised controlled trial (RCT) trialling a supported online self-management intervention for symptoms of fatigue, pain and urgency/incontinence in people with inflammatory bowel disease: the IBD-BOOST trial. The full methodology of the RCT can be found in the study protocol [43] (Clinical trial number: ISRCTN71618461, https://www.isrctn.com/; Registration date: 09/09/2019). This study received ethical approval from the NRES Research Ethics Committee & Health Research Authority (London - Surrey Research Ethics Committee/ 19/LO/0750). It was conducted in accordance with the principles of the Declaration of Helsinki. Online informed consent was obtained from all participants.

### Participants and procedure

In brief, participants were initially recruited for the RCT from the IBD-BOOST survey, a UK national survey [54]. These participants were recruited from IBD clinic hospital sites, the UK National IBD BioResource, the charity Crohn’s & Colitis UK or via social media. Participants were sent the survey in the post or by an email containing a link. If participants consented to be contacted for follow-up research, they were screened for eligibility prior to entering the BOOST trial. They received information sheets, and completed online consent and trial baseline questionnaires before they were randomised.

### Eligibility criteria

Eligibility criteria for participants were: being 18 years or older; living in England, Scotland, or Wales, having a diagnosis of IBD; having access to a computer or mobile device; and having self-scored one or more symptoms of fatigue, pain, or urgency/incontinence as having an impact on their quality of life of 5 or more on a 0–10 scale when completing the IBD-BOOST survey.

Participants were excluded if they were unable to give informed consent, had insufficient command of the English language for study procedures or demonstrated any ‘red flags’ (potentially serious issues, for example, new bleeding, rapid weight loss or vomiting that had not been previously reported to a healthcare practitioner). Additionally, for the purpose of this cross-sectional analysis, participants were excluded if they did not provide an FCP sample as part of the baseline assessment.

### Data

This study used data collected at baseline for an RCT, in accordance with GDPR guidelines and handled as per the Data Protection Act (2018). The data collection process for this baseline assessment included a questionnaire assessment (online or paper), as well as a postal stool sample sent to a central laboratory for measurement of FCP.

### Measures

A full list of the measures collected as part of the BOOST trial are reported elsewhere [43]. Listed below are measures that were used in this analysis collected as part of the trial baseline assessment:

- Self-reported disease activity (SRDA) of IBD disease was measured with the IBD-Control, an 8-item questionnaire [22] designed to measure disease control from the patient’s perspective over the last two weeks. Higher scores indicate better control and lower disease activity [22] (Cronbach α = 0.78).
- Objective disease activity was assessed with faecal calprotectin (FCP) [15] from stool samples. Calprotectin is a protein that can bind to calcium and zinc, typically presented in neutrophil cells [55]. FCP calprotectin is highly sensitive marker of inflammation in the gastrointestinal tract and a key indicator for an IBD diagnosis. Furthermore, it is used to monitor disease activity, guide treatment decisions, and predict relapse in IBD [15]. FCP was the primary clinical biomarker of disease severity used in the current study. It was analysed using the Diasorin Liaison XL Calprotectin assay, an in vitro diagnostic chemiluminescent immunoassay (CLIA), at King’s College Hospital, London.
- Depressive symptoms was assessed using the nine-item Patient Health Questionnaire– 9 (PHQ-9) [56]. Each item asks about the frequency of a depression symptom over the last two weeks and is responded to on a 4-point Likert Scale (0–3). The PHQ-9 has a scale range of 0–27, with higher scores indicating increased depressive symptoms [56]. Scores of 0–4, 5–9, 10–14, 15–29, and ≥ 20 on the PHQ-9 indicate levels of no, mild, moderate, moderately severe, and severe depressive symptoms respectively [56] (Cronbach α = 0.87).
- Data on health service use and employment were measured using a study-specific IBD Resource Use questionnaire. This instrument was developed within the BOOST programme, and covers primary and secondary care, investigations, medications, hospitalisations, employment and out-of-pocket expenses over the last 3 months. The questionnaire was found to be reliable and its online functionality was tested in a pilot study [57]. For the purposes of this analysis, primary care data (GP, and GP nurse visits) secondary care data (gastroenterologist, colorectal surgeon, pharmacist, IBD nurse and IBD advice visits), Accident and Emergency (A&E) visits, and hospitalisations were extracted. Composite scores for primary care, secondary care and total visits were calculated by summing the relevant visits to specific services. The economic indicators asked participants over the last 3 months (1) the number of sick leave days due to IBD and (2) the impact of IBD on productivity (0 = no effect on work, 10 = completely prevented me from working).

Sociodemographic variables (age, gender, ethnicity, education level, relationship status, employment) and additional clinical information (IBD subtype, body mass index (BMI)) were linked across from the IBD-BOOST Survey.

### Statistical analysis

Data were analysed with Stata (version 18, Windows), with questionnaires scored in accordance with original authors’ guidelines. Cronbach’s alpha was used to assess internal consistency and reliability. Descriptive statistics were computed for all variables using means and standard deviations and/or medians and ranges/interquartile ranges for continuous data, and frequencies and percentages for categorical data. Firstly, bivariate associations assessed relationships between depression and demographics, clinical covariates, visits to specific health services, composite health service visits (primary care, secondary care, total visits) and economic indicators (number of sick leave days, impact of IBD on productivity), as well as association between inflammation (FCP) and SRDA (IBD-Control). These associations were examined using independent samples t-tests for binary variables, Pearson’s for normally distributed continuous data, and Spearman’s rank for non-normal continuous data. Categorical variables were dummy coded and analysed using multiple linear regression models. For the associations between FCP and depression, sub-group analyses were run, splitting the sample firstly, by disease subtype (UC/other and CD) and secondly, by biochemical activity status (remission, FCP < 200 µg/g and flare, FCP ≥ 200 µg/g).

Next, a series of regression models were run. Firstly (for Aim 1), both objective (FCP) and subjective (IBD-Control) measures of IBD activity were inputted as linear predictors in a multiple linear regression model with depression as the outcome variable, whilst controlling for relevant sociodemographic and clinical factors. FCP has limited ability to detect small bowel inflammation, which is more common in Crohn’s Disease, therefore an interaction term of FCP and disease subtype (Crohn’s Disease coded as 1, Ulcerative Colitis coded as 0) was included. Variables were inputted in two steps: firstly, objective and subjective measures of IBD activity; next, key variables with significant associations with depression identified from the bivariate analyses and the FCP-subtype interaction term were entered. Secondly (for Aim 1), a multiple linear regression to explain variance in SRDA (IBD-Control) was conducted including FCP, disease subtype and the subtype-FCP interaction term as explanatory variables. Next for the analysis of health service and economic indicators (Aim 2), depression, FCP and SRDA were all inputted into multiple linear regressions to determine the variance explained for each outcome (primary care visits, secondary care visits, A&E visits, days taken off work due to IBD and impact of IBD on productivity at work). Outcomes were operationalised to be linear, instead of categorical, to increase statistical power [58], and to emulate previous research [47]. Composite scores were calculated for primary care visits (summing GP and GP nurse visits), secondary care visits (summing gastroenterologist, colorectal surgeon, pharmacist, IBD nurse and IBD advice visits) and total visits (summing primary and secondary care visits). A sensitivity analysis was conducted, additionally including the FCP-subtype interaction term. Standardised beta values, 95% confidence intervals and *p* values were reported in linear regressions. Significance level for all analyses was set to *p* < 0.05. In our sample, the minimum detectable effect (80% power, 5% significance level) was a *r* = 0.12.

## Results

### Descriptives

In total, 780 participants consented to participate in the trial and completed baseline surveys. A total of 181 participants did not have a value for FCP either because they had a stoma (*n* = 48) or because they did not return their sample (*n* = 133). This resulted in a sample of 599 participants who were mostly (66.44%) female, the majority had Crohn’s Disease (56.43%), and the majority were White (95.49%) with a mean age of 50.17 (SD = 14.17). FCP tests were performed a median of 30 days (IQR: 21, 44) after completion of the questionnaire. FCP was positively skewed, with a median value of 48 (IQR: 16–141.5). Using the standard cut-off of 200 µg/g, 19.03% of the sample were in biochemical flare. The majority (75.63%) of participants had clinically significant levels of depression (mild, moderate or severe), with a mean score of 9.01 (SD = 5.72). Based on IBD-Control cut-offs (≤ 13 indicate flare [22]), 76.29% self-reported that they were in flare. Full sample characteristics are reported in Table 1.

**Table 1:** Demographic and clinical characteristics of the sample and their associations with depression (*n* = 599)

Variable	*N* (%) / M(SD)	Depression M(SD)	Statistical test	β	95%CI	*P* value
**Demographic variables**						
*Gender*			F(2,596) = 0.44			0.641
Female	398 (66.44%)	9.08 (5.67)	*t* = 0.88	0.296	− 0.363, 0.954	0.378
Male	199 (33.22%)	8.90 (5.85)	*t* = 0.84	0.280	− 0.379, 0.939	0.404
Other / Prefer not to say	2 (0.33%)	5.50 (4.95)	-	-		
*Ethnicity*			F(5,593) = 1.96			0.083
Arab	2 (0.33%)	19.00 (5.66)	*t* = 2.09	0.103	0.306, 0.201	0.038
Asian or Asian British	14 (2.34%)	6.86 (5.50)	*t*=-0.59	-0.050	− 0.218, 0.118	0.556
Black or Black British	1 (0.17%)	6.00	*t*=-0.43	-0.020	− 0.109, 0.070	0.666
Mixed/multiple ethnic groups	6 (1.00%)	6.33 (3.72)	*t*=-0.66	-0.042	− 0.168, 0.084	0.512
White	572 (95.49%)	9.06 (5.73)	*t* = 0.11	0.011	− 0.379, 0.215	0.913
Other/unknown	4 (0.67%)	8.75 (4.03)	-			
*Age*	35.55 (12.67)		*r* =-0.194		− 0.270, − 0.116	< 0.001
*Employment*			F(5,593) = 9.40			< 0.001
Employed	336 (56.09%)	8.84 (5.28)	*t*=-2.42	-0.169	− 0.306, − 0.032	0.016
Retired	147 (24.54%)	7.50 (5.41)	*t*=-3.75	-0.247	− 0.377, − 0.118	< 0.001
Student	13 (2.17%)	10.54 (5.78)	*t*=-0.14	-0.006	− 0.092, 0.080	0.886
Unemployed due to illness disability	47 (7.85%)	12.43 (6.59)	*t* = 1.49	0.077	− 0.025, 0.179	0.138
Other (e.g., homemaker)	56 (9.35%)	10.79 (6.70)	-			
*Education*			F(2,594) = 1.87			0.156
Further or higher	457 (76.88%)		*t*=-1.77	-0.284	− 0.600, 0.031	0.078
School	131 (21.94%)		*t*=-1.51	-0.242	− 0.558, 0.073	0.131
No formal education	7 (1.17%)		-	-		
*Relationship status*			F(5,590) = 1.16			0.329
Married or civil partnership	338 (56.71%)		*t*=-1.79	-0.172	− 0.360, 0.017	0.074
Cohabiting	94 (15.77%)		*t*=-1.14	-0.088	− 0.240, 0.064	0.257
Divorced or separated	40 (6.71%)		*t*=-1.62	-0.099	− 0.219, 0.021	0.105
Widowed	15 (2.52%)		*t*=-1.48	-0.074	− 0.172, 0.024	0.140
Single	80 (13.42%)		*t*=-0.79	-0.058	− 0.204, 0.087	0.431
Not living with partner	29 (4.87%)		-			
**Clinical variables**						
*Diagnosis*			*t* = 1.928			0.054
Crohn’s Disease	338 (56.43%)	9.41 (5.83)				
Ulcerative Colitis and other diagnoses	261 (43.57%)	8.50 (5.55)				
*Body Mass Index (BMI)*			F(2,580) = 9.41			< 0.001
Underweight	22 (3.77%)	10.14 (6.49)	*t* = 1.92	0.080	− 0.002, 0.163	0.055
Overweight	342 (58.56%)	9.78 (5.85)	*t* = 4.23	0.178	0.095, 0.260	< 0.001
Healthy weight	219 (37.56%)	7.72 (5.15)	-			
*Faecal calprotectin* (median, IQR)	48, 16–141.5		*r* = 0.024		− 0.055, 0.103	0.561
*Faecal calprotectin categories*			*t* = -1.107			0.269
Biochemical remission (< 200 µg/g)	485 (80.97%)	8.88 (6.00)				
Biochemical flare (≥ 200 µg/g)	114 (19.03%)	9.54 (6.22)				
*Self-report disease activity (IBD-Control)*	9.00 (4.31)		*r* = -0.605		− 0.654, − 0.552	< 0.001
*Self-report disease activity categories*			*t* = -11.57			< 0.001
Inactive/Good control (IBD-Control > 13)	142 (23.71%)	4.62 (3.70)				
Active/Poor control (IBD-Control ≤ 13)	457 (76.29%)	10.37 (5.55)				
*Economic indicators*						
Number of sick leave days (median, IQR)	0 (0–1)		*r* = 0.378		0.286, 0.471	< 0.001
IBD impact on productivity	3.26 (2.34)		*r* = 0.460		0.373, 0.549	< 0.001
*Health service use*						
Gastroenterologist			*t* = -2.617			0.009
Yes	239 (39.90%)	9.76 (0.39)				
No	360 (60.10%)	8.51 (0.29)				
Colorectal Surgeon			*t* = -2.991			0.003
Yes	48 (8.04%)	11.38 (0.91)				
No	549 (91.96%)	8.81 (0.24)				
IBD nurse			*t* = -1.940			0.053
Yes	278 (46.41%)	9.50 (0.37)				
No	321 (53.59%)	8.59 (0.30)				
IBD advice helpline			*t* = -2.694			0.007
Yes	141 (23.58%)	10.15 (0.54)				
No	457 (76.42%)	8.67 (0.26)				
A&E attendance			*t* = -2.680			0.008
Yes	27 (4.52%)	11.89 (0.24)				
No	571 (95.48%)	8.88 (1.33)				
GP			*t* = -4.707			< 0.001
Yes	175 (29.26%)	10.70 (0.47)				
No	423 (70.74%)	8.32 (0.26)				
GP Nurse			*t* = -2.643			0.008
Yes	103 (17.22%)	10.37 (0.58)				
No	495 (82.78%)	8.74 (0.25)				
Pharmacist			*t* = -3.540			< 0.001
Yes	108 (18.09%)	10.76 (0.54)				
No	489 (81.91%)	8.63 (0.26)				
Hospitalised			*t* = -1.672			0.095
Yes	17 (2.84%)	11.29 (2.06)				
No	582 (97.16%)	8.94 (0.23)				
Sick leave due to IBD*			*t* = -6.623			< 0.001
Yes	107 (32.62%)	11.38 (0.49)				
No	221 (67.38%)	7.54 (0.33)				
Total visits (median, IQR)	2 (0–5)		*r* = 0.260		0.181, 0.331	< 0.001
Primary care visits (median, IQR)	0 (0–1)		*r* = 0.205		0.126, 0.283	< 0.001
Secondary care visits (median, IQR)	2 (0–4)		*r* = 0.207		0.131, 284	< 0.001
Times hospitalised (median, range)	0 (0–3)		*r* = 0.034		− 0.057, 0.124	0.416
Days in hospital (median, range)	0 (0–38)		*r* = 0.036		− 0.058, 0.126	0.414
*Depression (PHQ-9)*	9.01 (5.72)		-			-
*Depression categories*			-			-
None (PHQ-9 < 5)	146 (24.37%)	-				
Mild (5 ≤ PHQ-9 ≤ 9)	206 (34.39%)	-				
Moderate-Severe (PHQ-9 > 9)	247 (41.24%)	-				

* denotes *p* value ≤ 0.05; ** denotes *p* value ≤ 0.01; *** denotes *p* value ≤ 0.001

A&E = accident and emergency, IBD = inflammatory bowel disease, IQR = interquartile range, PHQ-9 = Patient Health Questionnaire-9

### Depression and relationships with objective inflammation and SRDA

Table 1 shows the associations between depression and key sociodemographic variables, and other variables identified to be influential with depression and inflammation. Depression was significantly associated with employment status (F(5,593) = 9.40, *p* < 0.001), with retirees demonstrating the lowest level of depression and those unemployed demonstrating the highest level of depression. Additionally, depression was associated with BMI (F(2,580) = 9.41, *p* < 0.001), with overweight BMI positively associated with depression. Depression was negatively associated with age (*r*=-0.194, *p* < 0.001). Depression was associated with SRDA, with higher depression related to more severe SRDA (*r*=-0.605, *p* < 0.001, see Supplementary Material 1 for scatter plot). There was a non-significant relationship with FCP (*r* = 0.038, *p* = 0.252, See Supplementary Material 2 for scatter plot). Two sub-group analyses were performed. Depression and FCP associations remained nonsignificant when the sample was split by disease subtype (UC: *r* = 0.091, *p* = 0.144; CD: *r*=-0.028, *p* = 0.603), and by biochemical remission/flare status (remission, FCP < 200: *r*=-0.002, *p* = 0.974; flare, FCP ≥ 200: *r* = 0.032, *p* = 0.738).

Table 2 shows the results of the multivariate analyses. The model including SRDA (IBD-Control) and objective inflammation (FCP) (Step 1) explained 36.6% of the variance in depression (F(2,596) = 175.1, *p* < 0.001). SRDA was a significant predictor (β=-0.611, *p* < 0.001, 95%CI: − 0.676, − 0.546), but FCP was non-significant (β=-0.040, *p* = 0.227, 95%CI: − 0.105, 0.025). When relevant demographic variables (age, gender, employment status), BMI, disease subtype and the subtype-FCP interaction term were added into the model (Step 2), the model explained 40.68% of the variance (F(12,570) = 34.26, *p* < 0.001). SRDA remained a significant predictor of depression (β=-0.573, *p* < 0.001, 95%CI: − 0.639, − 0.507) and FCP remained nonsignificant (β=-0.076, *p* = 0.102, 95%CI: − 0.166, 0.015). Age additionally negatively predicted depression (β=-0.151, *p* = 0.001, 95%CI: − 0.238, − 0.063). Being unemployed significantly predicted depression (β = 0.125, *p* < 0.001, 95%CI: 0.060, 0.189), as did being overweight (β = 0.213, *p* = 0.002, 95%CI: 0.079, 0.346). Neither disease subtype nor the subtype-FCP interaction term were significant.

**Table 2:** Multivariate regression analysis of predictors for depression

		Step 1				Step 2	
	β	*p*	95%CI		β	*p*	95%CI
SRDA (IBD-Control)	-0.611	< 0.001	− 0.676, − 0.546		-0.573	< 0.001	− 0.639, − 0.507
FCP	-0.040	0.227	− 0.105, 0.025		-0.076	0.102	− 0.166, 0.015
Age					-0.150	0.001	− 0.238, − 0.063
Gender (reference = female)					0.063	0.056	− 0.002, 0.127
Employment (reference = employed)							
Retired					0.012	0.794	− 0.075, 0.098
Student					0.037	0.262	− 0.028, 0.101
Unemployed					0.125	< 0.001	0.060, 0.190
Other					0.064	0.057	− 0.002, 0.131
BMI (reference = healthy weight)							
Underweight					0.001	0.970	− 0.064, 0.067
Overweight					0.105	0.002	0.039, 0.171
Disease subtype (reference = UC)					-0.001	0.967	− 0.068, 0.071
Disease subtype interaction term					0.053	0.276	− 0.042, 0.146

*Note*. BMI = body mass index, FCP = faecal calprotectin, IBD = inflammatory bowel disease, SRDA = self-reported disease activity, UC = Ulcerative Colitis

### The relationship between objective inflammation and SRDA

SRDA, measured by the IBD-Control, showed a significant, weak, negative correlation with intestinal inflammation as measured by FCP (*r*=-0.165, *p* < 0.001, 95%CI: − 0.240, − 0.089; see Supplementary Material 3 for scatterplot). A multivariate regression model of FCP, disease subtype and the subtype-FCP interaction term significantly predicted SRDA (F(3,595) = 6.85) explaining 2.85% of the variance. Only FCP was a significant predictor of SRDA (β=-0.204, *p* = 0.001, 95%CI: − 0.319, − 0.088). The subtype-FCP interaction term was not a significant predictor, indicating the relationship between FCP and SRDA did not significantly differ between Crohn’s Disease and Ulcerative Colitis (β=-0.112, *p* = 0.355, 95%CI: − 0.125, 0.348).

### Health service and economic indicators and relationships with depression, objective inflammation and SRDA

Test statistics of bivariate analyses (Pearson’s r, Spearman’s rho, t values) are summarised in Table 1. Depression was significantly associated with primary care visits (composite variable as well as visits to GP, GP nurse and pharmacist), secondary care visits (composite variable as well as visits to gastroenterologist, colorectal surgeon, IBD nurse, IBD advice), A&E attendance, sick leave days and impact of IBD on productivity, but not with times hospitalised or with days in hospital.

In the multivariate analyses, multiple linear regressions including depression, FCP and SRDA, significantly predicted all health service use outcomes (See Table 3). Depression was the only significant predictor in the models for primary care visits and accident and emergency visits, with models explaining 6.4% and 1.9%, respectively. The model explained 12.3% of the variance for secondary care visits and 14.0% for total visits, with depression, FCP and SRDA all emerging as significant predictors. Although depression was significantly associated with days taken off work in the bivariate analysis (*r* = 0.378, *p* < 0.001), when other factors were controlled, depression was rendered non-significant and SRDA was the only significant predictor of sick leave days (β=-0.149, 95%CI: − 0.286, − 0.012, *p* = 0.033), with the model explaining 2.6% of the variance. The model for impact of IBD on work productivity explained 31.7% of the variance, with depression and SRDA emerging as significant predictors. Sensitivity analyses for each health service/economic outcome were conducted additionally including disease subtype and the subtype-FCP interaction term. The size and significance of the betas did not change substantially from the original analysis. Crohn’s Disease was predictive of primary care visits and total visits. The subtype-FCP interaction term was significant for primary care visits, secondary care visits and total visits, indicating a more pronounced effect of FCP on usage in Ulcerative Colitis patients rather than Crohn’s Disease patients (See Supplementary Material 4).

**Table 3:** Results of regression models for health service (*n* = 599), sick leave days (*n* = 327) and impact of IBD on productivity at work (*n* = 336) with depression, inflammation and self-reported disease activity entered as predictor variables

	Adjusted *r* Square	Model significance	Depression (PHQ-9) β (95%CI)	Faecal calprotectin β (95%CI)	SRDA (IBD-Control) β (95%CI)
Primary care visits	0.064***	F(3,595) = 14.61	0.192 (0.094, 0.290)***	0.022 (-0.057,0.101)	− 0.093 (-0.192, 0.006)
Secondary care visits	0.123***	F(3,595) = 29.07	0.130 (0.035, 0.224)**	0.169 (0.092, 0.246)***	− 0.197 (-0.293, − 0.101)***
A&E visits	0.019**	F(3,595) = 4.92	0.101 (0.001,0.201)*	0.056 (-0.025, 0.137)	− 0.053 (-0.155, 0.048)
Total visits	0.140***	F(3,595) = 33.42	0.180 (0.086, 0.274)***	0.144 (0.069, 0.220)***	− 0.193 (-0.289, − 0.098)***
Sick leave days ^Ψ^	0.026**	F(3,323) = 3.95	0.058 (-0.086, 0.203)	0.023 (-0.083, 0.129)	− 0.149 (-0.286, − 0.012)*
Impact on productivity ^Ψ^	0.317***	F(3,332) = 52.77	0.236 (0.118, 0.354)***	0.038 (-0.049, 0.126)	− 0.413 (-0.526, − 0.300)***

*Note*. *** indicates *p* < 0.001, ** *p* < 0.01, * *p* < 0.05

^Ψ^ analysis only conducted in proportion of sample in employment (i.e., not retired or not working for reasons unrelated to IBD)

A&E = accident and emergency, IBD = inflammatory bowel disease, PHQ-9 = Patient Health Questionnaire-9, SRDA = self-reported disease activity

## Discussion

This analysis included 599 IBD patients, finding that 76% had at least mild depression. Notably, 81% were in biochemical remission (FCP < 200 µg/g), but 76% indicated poor self-reported control of IBD (IBD-Control ≤ 13). Higher self-reported depression was associated with greater SRDA (lower IBD-Control). Sociodemographic and clinical characteristics associated with depression included older age, being unemployed and being overweight based on BMI. Depression was not significantly associated with inflammation levels, as measured by FCP. A regression model including both objective inflammation and IBD disease control explained 36.8% of the variance in depression, however only SRDA was a significant predictor, which remained significant after relevant variables were controlled. Although SRDA was significantly negatively associated with inflammation, the correlation was weak. Depression was an independent predictor of visits to primary care, secondary care, and A&E, as well as impact of IBD on productivity.

Contrary to our prediction, FCP was not related to depression. However, our findings corroborated previous research linking anxiety and depression with gastrointestinal symptoms, but not with inflammation [6]. This contradicts the conceptual framework of the gut-brain axis, which proposes depression as having pro-inflammatory consequences and inflammatory disease activity impacting psychological processes [7, 8, 11, 14, 46]. Methodological issues may explain our null findings. For instance, most of the sample were in biochemical remission, with a median lag of 30 days between sample collection and questionnaire completion. However, our sub-group analysis of those in biochemical flare was also yielded nonsignificant results. This accords with other studies [26, 59–62], including those reporting a broader range of inflammation (e.g., FCP median = 132.7, IQR: 47, 507 [6]) that also found no contemporaneous associations between depression and FCP. Studies that do find significant associations between FCP are often limited by small sample sizes, inadequate inclusion of control variables (for instance, subtype, demographics or self-reported disease activity) and/or failure to adjust for multiple statistical comparisons [63–66]. One study found a positive weak association (*r* = 0.142, *p* = 0.04) between FCP and depression in 193 CD patients but not in UC [66]. However, this study did not control for clinical disease activity, and it is unclear why there was no relationship with depression in the UC sub-group [66], where FCP should have greater salience as a biomarker [51–53]. The null findings, and some of the inconsistencies observed in previous research, may represent the complexity and individual variability within the gut-brain axis, and the mechanisms through which peripheral inflammation drives central inflammation and depression [67]. Peripheral inflammation does not cause depression in all individuals [68], and factors such as the blood brain barrier [68] or microbiome composition and function [69–71] have been shown to moderate the relationship between psychological factors and inflammation. Conversely, associations have been found with depression and other measures of inflammation in IBD, such as mucosal inflammation as measured by endoscopy, proinflammatory cytokines, monocyte and macrophage concentrations [72–76]. Future studies would also benefit from using multiple inflammatory markers to clarify these relationships within the gut-brain axis, as well as longitudinal study designs so causality can be inferred.

Moreover, given the heterogeneity of IBD, it may be that depression has different gut-brain implications between individuals, based on environmental, genetic, pharmacological or behavioural differences [77]. Therefore, the eligibility criterion of those with symptom burden with a greater impact on quality of life may have led to overrepresentation of people whose depression and related psychological factors have a larger influence on symptom perception. This may preclude the inclusion of individuals with greater vulnerability to bidirectional neuroinflammatory pathways where depression and gut inflammation have stronger associations, irrespective of symptoms. Future research should attempt to use representative samples and stratify people living with IBD into different ‘gut-brain’ profiles [78] to assess individual differences in gut-brain mechanisms.

Furthermore, the recruitment strategy selecting patients where symptoms have high impact on quality of life may have influenced our findings that SRDA weakly correlated with objective inflammatory disease activity, even when differential relationships of inflammation and SRDA between disease subtypes were investigated. However, this finding is strongly corroborated by other studies, where self-reported measures of symptoms are more strongly associated with psychological factors, including depression [6], than objective inflammatory disease markers [26–29]. Many neurobiological and cognitive processes may underly the relationship between depression and symptoms [79]. Psychological factors may perpetuate or exacerbate symptom perception and reporting, potentially contributing to the high symptom burden [80], despite low levels of inflammation. For instance, in IBD, illness perceptions, stress, anxiety and depression are routinely associated with IBD symptom reports, disability and health-related quality of life [30, 81–85]. Specific cognitive biases that may contribute to symptoms in the absence of inflammation include (i) somatic hypervigilance [86, 87], whereby excessive focus on bodily sensations predisposes patients to misinterpret sensations as meaningful symptoms [86, 87]; (ii) confirmation bias [88], where prior experiences prime patients to attribute mild symptoms to be more severe, overlooking alternative explanations; and (iii) catastrophising, a cognitive distortion where patients anticipate the worst outcome from their symptoms which can increase symptom perception [30, 89].

Relatedly, both depression and SRDA independently predicted most healthcare usage and economic indicators, even when objective inflammation was controlled. PROMs are increasingly used in IBD clinical management and as efficacy measures in IBD clinical trials [90]. This has implications for clinical practice as although severe symptoms warrant medical attention, biomedical treatments may not be wholly appropriate if symptoms are weakly related to inflammation. This is supported by qualitative research findings that gastroenterologists prefer to focus on objective disease activity and struggle to address functional symptoms in the absence of inflammation and clear underlying pathology [91]. Moreover, depression as an independent predictor, may directly and indirectly affect healthcare usage; indirectly by influencing symptoms such as pain or fatigue [79, 92] and exacerbating severity [30] and directly through increase help-seeking behaviour due to feeling unable to cope with disease or treatment [93]. Given that the gut-brain axis is bidirectional, inflammation may increase both symptoms and depression, which would be related to both disease severity and healthcare usage [13]. However, in this study inflammation, as measured by FCP, was not associated with depression, and only showed a small effect [94] with secondary care visits, but was nonsignificant with other indicators of healthcare usage. Such relationships may emerge if inflammation, disease activity and depression in longitudinal investigations or if other inflammatory markers were measured [72], particularly due to the limitation of FCP in detecting small bowel inflammation. Regardless, our findings emphasise the need for a holistic approach to healthcare that considers both mental and physical health factors to optimise resource allocation within IBD treatment [95]. Given that substantial variance in healthcare usage was explained by psychological factors, treating depression may help to reduce costs for health services [96].

### Strengths and limitations

This study used a large sample size and objective measures of inflammation to assess the relationships between depression and disease activity in people living with IBD. Analyses were adequately controlled, and the relationships with health service use and economic indicators were additionally explored.

This study was limited in several ways. (1) It used cross-sectional data, so causality cannot be determined. Previous evidence suggests bidirectionality between depression and negative health outcomes (e.g., hospitalisations) [46], so the associations in the current study may additionally reflect these relationships. The PHQ-9 and IBD-Control ask about symptoms over the last two weeks, whereas, the Resource Use questionnaire collects data from previous 3 months. However, given that our findings do not contradict existing literature [44–47], these approximations appear to have been adequate. (2) Study participants had high rates of clinically relevant depression (75.6%), poor IBD control (76.3%) and biochemical remission (80.1%). Therefore, relationships between inflammation, depression and disease activity may be different when participants with higher levels of inflammation and/or better IBD control are investigated. (3) Relatedly, although the study originally recruited from NHS hospitals, due to the COVID-19 pandemic, recruitment shifted online to access community samples. As such, participants self-selected, possibly leading to unrepresentative samples, thus limiting the generalisability of the findings. (4) Despite the IBD resource use questionnaire demonstrating good test re-test reliability [57], it has yet to be fully validated and healthcare usage data may be susceptible to recall bias that would impact findings. (5) Although medications may impact pain perception independent of inflammation (for instance, antidepressants can reduce pain [97, 98]; steroids can contribute to mood alterations, fatigue and gastrointestinal discomfort [99]), it was beyond the scope of the study to investigate medications as confounding variables.

## Conclusions

This study demonstrated that higher depression was associated with greater SRDA, but not with intestinal inflammation. Moreover, SRDA only weakly correlated with FCP. Depression and SRDA appear to independently explain variance in healthcare usage, emphasising the need for holistic approaches to IBD treatment that address both mental and physical health. Future research should utilise longitudinal data to understand causal relationships between these variables and their impact on healthcare usage and investigate relationships between depression and other inflammatory biomarkers.

## Electronic supplementary material

Below is the link to the electronic supplementary material.

Supplementary Material 1

## Data Availability

The data that support the findings of this study are available from the corresponding author on reasonable request subject to appropriate data sharing permissions.

## Abbreviations

A&E: Accident and emergency
BMI: Body mass index
CD: Crohn’s disease
CI: Confidence interval
CLIA: ChemiLuminescent immunoassay
CRP: C-Reactive protein
FCP: Faecal calprotectin
GP: General practitioner
HPA: Hypothalamic-pituitary-adrenal
IBD: Inflammatory bowel disease
NHS: National health service
NIHR: National institute of health research
PHQ-9: Patient health questionnaire-9
PROMs: Patient-reported outcome measures
RCT: Randomised controlled trial
SRDA: Self-reported disease activity
UC: Ulcerative Colitis

## References

[1] Baumgart DC, Carding SR. Inflammatory bowel disease: cause and immunobiology. Lancet (London England). 2007;369(9573):1627–40. 17499605 10.1016/S0140-6736(07)60750-8

[2] Neuman MG. Immune dysfunction in inflammatory bowel disease. Translational Research: J Lab Clin Med. 2007;149(4):173–86. 10.1016/j.trsl.2006.11.009 17383591

[3] Lichtenstein GR, Loftus EV, Isaacs KL, Regueiro MD, Gerson LB, Sands BE. ACG clinical guideline: management of Crohn’s disease in adults. Official J Am Coll Gastroenterology| ACG. 2018;113(4):481–517. 10.1038/ajg.2018.27 29610508

[4] Srinath A, Young E, Szigethy E. Pain management in patients with inflammatory bowel disease: translational approaches from bench to bedside. Inflamm Bowel Dis. 2014;20(12):2433–49. 25208108 10.1097/MIB.0000000000000170

[5] Barberio B, Zamani M, Black CJ, Savarino EV, Ford AC. Prevalence of symptoms of anxiety and depression in patients with inflammatory bowel disease: a systematic review and meta-analysis. Lancet Gastroenterol Hepatol. 2021;6(5):359–70. 33721557 10.1016/S2468-1253(21)00014-5

[6] Mules TC, Swaminathan A, Hirschfeld E, Borichevsky G, Frampton C, Day AS, et al. The impact of disease activity on psychological symptoms and quality of life in patients with inflammatory bowel disease—results from the stress, anxiety and depression with Disease Activity (SADD) Study. Aliment Pharmacol Ther. 2022;55(2):201–11. 34587655 10.1111/apt.16616

[7] Moulton CD, Pavlidis P, Norton C, Norton S, Pariante C, Hayee B, et al. Depressive symptoms in inflammatory bowel disease: an extraintestinal manifestation of inflammation? Clin Exp Immunol. 2019;197(3):308–18. 30762873 10.1111/cei.13276 PMC6693970

[8] Ge L, Liu S. Psychological stress in inflammatory bowel disease: psychoneuroimmunological insights into bidirectional gut–brain communications. Front Immunol. 2022;13:1016578. 36275694 10.3389/fimmu.2022.1016578 PMC9583867

[9] Günther C, Rothhammer V, Karow M, Neurath MF, Winner B. The gut-brain axis in inflammatory bowel disease—current and future perspectives. Int J Mol Sci. 2021;22(16):8870. 34445575 10.3390/ijms22168870 PMC8396333

[10] Craig CF, Filippone RT, Stavely R, Bornstein JC, Apostolopoulos V, Nurgali K. Neuroinflammation as an etiological trigger for depression comorbid with inflammatory bowel disease. J Neuroinflammation. 2022;19(1):4. 34983592 10.1186/s12974-021-02354-1 PMC8729103

[11] Miller AH, Maletic V, Raison CL. Inflammation and its discontents: the role of cytokines in the pathophysiology of major depression. Biol Psychiatry. 2009;65(9):732–41. 19150053 10.1016/j.biopsych.2008.11.029 PMC2680424

[12] Sanchez-Munoz F, Dominguez-Lopez A, Yamamoto-Furusho JK. Role of cytokines in inflammatory bowel disease. World J Gastroenterol. 2008;14(27):4280–8. 18666314 10.3748/wjg.14.4280 PMC2731177

[13] Fairbrass KM, Gracie DJ, Ford AC. Relative contribution of disease activity and psychological health to prognosis of inflammatory bowel disease during 6.5 years of longitudinal follow-up. Gastroenterology. 2022;163(1):190–203. e5. 35339461 10.1053/j.gastro.2022.03.014

[14] Seaton N, Hudson J, Harding S, Norton S, Mondelli V, Jones AS et al. Do interventions for mood improve inflammatory biomarkers in inflammatory bowel disease? A systematic review and meta-analysis. EBioMedicine. 2024;100. 10.1016/j.ebiom.2023.104910 PMC10878994 38272759

[15] Pathirana WGW, Chubb SP, Gillett MJ, Vasikaran SD. Faecal calprotectin. Clin Biochemist Reviews. 2018;39(3):77. PMC6370282 30828114

[16] Gracie DJ, Hamlin PJ, Ford AC. The influence of the brain–gut axis in inflammatory bowel disease and possible implications for treatment. Lancet Gastroenterol Hepatol. 2019;4(8):632–42. 31122802 10.1016/S2468-1253(19)30089-5

[17] Mikocka-Walus A, Knowles SR, Keefer L, Graff L. Controversies revisited: a systematic review of the comorbidity of depression and anxiety with inflammatory bowel diseases. Inflamm Bowel Dis. 2016;22(3):752–62. 26841224 10.1097/MIB.0000000000000620

[18] Hopkins CW, Moulton CD. Is it premature to advocate systematic psychological screening for patients with inflammatory bowel disease? Inflamm Bowel Dis. 2016;22(6):E19–20. 27167573 10.1097/MIB.0000000000000820

[19] Harvey R, Bradshaw J. A simple index of Crohn’s-disease activity. Lancet. 1980;315(8167):514. 10.1016/s0140-6736(80)92767-1 6102236

[20] Van Hees P, Van Elteren P, Van Lier H, Van Tongeren J. An index of inflammatory activity in patients with Crohn’s disease. Gut. 1980;21(4):279–86. 7429289 10.1136/gut.21.4.279 PMC1419605

[21] Walmsley RS, Ayres RC, Pounder RE, Allan RN. A simple clinical colitis activity index. Gut. 1998;43(1):29–32. 9771402 10.1136/gut.43.1.29 PMC1727189

[22] Bodger K, Ormerod C, Shackcloth D, Harrison M. Development and validation of a rapid, generic measure of disease control from the patient’s perspective: the IBD-control questionnaire. Gut. 2014;63(7):1092–102. 24107590 10.1136/gutjnl-2013-305600 PMC4078750

[23] Gebeyehu GG, Taylor F, Dobson L, Cummings JRF, Bloom S, Kennedy NA, et al. Validation of the IBD-Control Questionnaire across different sociodemographic and clinical subgroups: secondary analysis of a nationwide electronic survey. J Crohns Colitis. 2024;18(2):275–85. 37706542 10.1093/ecco-jcc/jjad147 PMC10896631

[24] Fletcher J, Cooper SC, Swift A. Patient-reported outcomes in inflammatory bowel disease: a measurement of effect in research and clinical care. Gastroenterol Insights. 2021;12(2):225–37.

[25] Kim AH, Roberts C, Feagan BG, Banerjee R, Bemelman W, Bodger K, et al. Developing a Standard Set of patient-centred outcomes for inflammatory bowel Disease—an International, cross-disciplinary Consensus. J Crohn’s Colitis. 2017;12(4):408–18. 10.1093/ecco-jcc/jjx161 29216349

[26] Gracie DJ, Williams CJ, Sood R, Mumtaz S, Bholah MH, Hamlin PJ, et al. Poor correlation between clinical disease activity and mucosal inflammation, and the role of psychological comorbidity, in inflammatory bowel disease. Am J Gastroenterol. 2016;111(4):541–51. 27002800 10.1038/ajg.2016.59

[27] Coates MD, Binion DG. Silent inflammatory bowel disease. Crohn’s Colitis 360. 2021;3(3):otab059. 34805984 10.1093/crocol/otab059 PMC8600959

[28] Click B, Vargas EJ, Anderson AM, Proksell S, Koutroubakis IE, Ramos Rivers C, et al. Silent Crohn’s disease: asymptomatic patients with elevated C-reactive protein are at risk for subsequent hospitalization. Inflamm Bowel Dis. 2015;21(10):2254–61. 26197446 10.1097/MIB.0000000000000516

[29] Falvey JD, Hoskin T, Meijer B, Ashcroft A, Walmsley R, Day AS, et al. Disease activity assessment in IBD: clinical indices and biomarkers fail to predict endoscopic remission. Inflamm Bowel Dis. 2015;21(4):824–31. 25738372 10.1097/MIB.0000000000000341

[30] Sweeney L, Moss-Morris R, Czuber-Dochan W, Meade L, Chumbley G, Norton C. Systematic review: psychosocial factors associated with pain in inflammatory bowel disease. Aliment Pharmacol Ther. 2018;47(6):715–29. 29359343 10.1111/apt.14493

[31] Binion DG. Silent Crohn’s disease. Gastroenterol Hepatol. 2014;10(8):510. PMC5566193 28845142

[32] Fairbrass KM, Costantino SJ, Gracie DJ, Ford AC. Prevalence of irritable bowel syndrome-type symptoms in patients with inflammatory bowel disease in remission: a systematic review and meta-analysis. Lancet Gastroenterol Hepatol. 2020;5(12):1053–62. 33010814 10.1016/S2468-1253(20)30300-9

[33] Wieser MJ, Pauli P. Chapter 1 - neuroscience of Pain and emotion. In: al’Absi M, Flaten MA, editors. Neuroscience of Pain, stress, and emotion. San Diego: Academic; 2016. pp. 3–27.

[34] Nakao M, Barsky AJ. Clinical application of somatosensory amplification in psychosomatic medicine. Biopsychosoc Med. 2007;1:17. 17925010 10.1186/1751-0759-1-17 PMC2089063

[35] Eller-Smith OC, Nicol AL, Christianson JA. Potential mechanisms underlying Centralized Pain and emerging therapeutic interventions. Front Cell Neurosci. 2018;12:35. 29487504 10.3389/fncel.2018.00035 PMC5816755

[36] Rusu AC, Gajsar H, Schlüter M-C, Bremer Y-I. Cognitive biases toward pain: implications for a neurocognitive processing perspective in chronic pain and its interaction with depression. Clin J Pain. 2019;35(3):252–60. 30499835 10.1097/AJP.0000000000000674

[37] Sheng J, Liu S, Wang Y, Cui R, Zhang X. The link between Depression and Chronic Pain: neural mechanisms in the brain. Neural Plast. 2017;2017(1):9724371. 28706741 10.1155/2017/9724371 PMC5494581

[38] Torta R, Ieraci V, Zizzi F. A review of the emotional aspects of neuropathic pain: from comorbidity to co-pathogenesis. Pain Therapy. 2017;6:11–7. 29178035 10.1007/s40122-017-0088-z PMC5701895

[39] Jones AS, Harding S, Seaton N, Hudson JL, Duff A, Wroe A, et al. A real-world longitudinal study implementing digital screening and treatment for distress in inflammatory bowel disease (IBD): the COMPASS-IBD study protocol. Contemp Clin Trials. 2024;145:107658. 10.1016/j.cct.2024.107658 39121990

[40] Sweeney L, Moss-Morris R, Czuber-Dochan W, Norton C. Pain management in inflammatory bowel disease: feasibility of an online therapist-supported CBT-based self-management intervention. Pilot Feasibility Stud. 2021;7(1):1–15. 33863398 10.1186/s40814-021-00829-9 PMC8050888

[41] Colombel J-F, Shin A, Gibson PR. AGA clinical practice update on functional gastrointestinal symptoms in patients with inflammatory bowel disease: expert review. Clin Gastroenterol Hepatol. 2019;17(3):380–90. e1. 30099108 10.1016/j.cgh.2018.08.001 PMC6581193

[42] Norton C, Czuber-Dochan W, Artom M, Sweeney L, Hart A. Systematic review: interventions for abdominal pain management in inflammatory bowel disease. Aliment Pharmacol Ther. 2017;46(2):115–25. 28470846 10.1111/apt.14108

[43] Norton C, Syred J, Kerry S, Artom M, Sweeney L, Hart A, et al. Supported online self-management versus care as usual for symptoms of fatigue, pain and urgency/incontinence in adults with inflammatory bowel disease (IBD-BOOST): study protocol for a randomised controlled trial. Trials. 2021;22(1):1–18. 34344432 10.1186/s13063-021-05466-4 PMC8329619

[44] Wong JJ, Sceats L, Dehghan M, Wren AA, Sellers ZM, Limketkai BN, et al. Depression and Health Care Use in patients with inflammatory bowel disease. J Crohn’s Colitis. 2018;13(1):19–26. 10.1093/ecco-jcc/jjy145 30256923

[45] Click B, Ramos Rivers C, Koutroubakis IE, Babichenko D, Anderson AM, Hashash JG, et al. Demographic and clinical predictors of high healthcare use in patients with inflammatory bowel disease. Inflamm Bowel Dis. 2016;22(6):1442–9. 26950309 10.1097/MIB.0000000000000763 PMC4868776

[46] Fairbrass KM, Lovatt J, Barberio B, Yuan Y, Gracie DJ, Ford AC. Bidirectional brain–gut axis effects influence mood and prognosis in IBD: a systematic review and meta-analysis. Gut. 2022;71(9):1773–80. 10.1136/gutjnl-2021-325985 34725197

[47] Irving P, Barrett K, Nijher M, de Lusignan S. Prevalence of depression and anxiety in people with inflammatory bowel disease and associated healthcare use: population-based cohort study. BMJ Ment Health. 2021;24(3):102–9. 10.1136/ebmental-2020-300223 PMC8311072 33785498

[48] Gracie DJ, Guthrie EA, Hamlin PJ, Ford AC. Bi-directionality of brain–gut interactions in patients with inflammatory bowel disease. Gastroenterology. 2018;154(6):1635–46. e3. 29366841 10.1053/j.gastro.2018.01.027

[49] Clough J, Colwill M, Poullis A, Pollok R, Patel K, Honap S. Biomarkers in inflammatory bowel disease: a practical guide. Th Adv Gastroenterol. 2024;17:17562848241251600. 10.1177/17562848241251600 PMC11085009 38737913

[50] D’Haens G, Ferrante M, Vermeire S, Baert F, Noman M, Moortgat L, et al. Fecal calprotectin is a surrogate marker for endoscopic lesions in inflammatory bowel disease. Inflamm Bowel Dis. 2012;18(12):2218–24. 22344983 10.1002/ibd.22917

[51] Cerrillo E, Beltrán B, Pous S, Echarri A, Gallego JC, Iborra M, et al. Fecal calprotectin in ileal Crohn’s disease: relationship with magnetic resonance enterography and a pathology score. Inflamm Bowel Dis. 2015;21(7):1572–9. 26052967 10.1097/MIB.0000000000000404

[52] Kopylov U, Nemeth A, Koulaouzidis A, Makins R, Wild G, Afif W, et al. Small bowel capsule endoscopy in the management of established Crohn’s disease: clinical impact, safety, and correlation with inflammatory biomarkers. Inflamm Bowel Dis. 2015;21(1):93–100. 25517597 10.1097/MIB.0000000000000255

[53] Stawczyk-Eder K, Eder P, Lykowska-Szuber L, Krela-Kazmierczak I, Klimczak K, Szymczak A, et al. Is faecal calprotectin equally useful in all Crohn’s disease locations? A prospective, comparative study. Archives Med Sci. 2015;11(2):353–61. 10.5114/aoms.2014.43672 PMC4424241 25995752

[54] Roukas C, Miller L, Cléirigh Büttner F, Hamborg T, Stagg I, Hart A et al. Impact of pain, fatigue and bowel incontinence on the quality of life of people living with inflammatory bowel disease: a UK cross-sectional survey. United Eur Gastroenterol J. 2024. 10.1002/ueg2.12668 PMC7616848 39425758

[55] Fagherol M, Andersson K, Naess-Andresen C, Brandtzaeg P, Dale I. Calprotectin (the L1 leucocyte protein). In: Smith VL, Dedman JR, editors. Stimulus response coupling: the role of intracellular calcium-binding proteins. Boca Raton: CRC; 1990.

[56] Kroenke K, Spitzer RL. The PHQ-9: a new depression diagnostic and severity measure. Psychiatric Annals. 2002;32(9):509–15.

[57] Roukas C, Syred J, Gordeev VS, Norton C, Hart A, Mihaylova B. Development and test–retest reliability of a new, self-report questionnaire assessing healthcare use and personal costs in people with inflammatory bowel disease: the inflammatory bowel Disease Resource Use Questionnaire (IBD-RUQ). Frontline Gastroenterol. 2023;14(1):59–67. 36561790 10.1136/flgastro-2022-102182 PMC9763637

[58] Heidel RE. Causality in statistical power: Isomorphic Properties of Measurement, Research Design, Effect size, and Sample Size. Scientifica (Cairo). 2016;2016:8920418. 27073717 10.1155/2016/8920418 PMC4814708

[59] Baig BJ. Inflammatory bowel disease, steroids and affective disorder. 2019.

[60] Gounden S, Brett L, Mcivor C, editors. Characterizing Kessler Psychological Distress Scale scores and psychosocial stressors in an Australian inflammatory bowel disease cohort. WILEY 111 RIVER ST, HOBOKEN 07030– 5774, NJ USA: JOURNAL OF GASTROENTEROLOGY AND HEPATOLOGY; 2020.

[61] Cohen-Lyons D, Nazarian A, Griller N, Kandel G, Grover SC, Kim Y-I, et al., editors.The Rapid Fecal Calprotectin Test in IBD Patients in Clinical Remission: Correlations With Activity Index, IBS-Like Symptoms, Anxiety, Depression and Quality of Life. Gastroenterology;2016: WB SAUNDERS CO-ELSEVIER INC 1600 JOHN F KENNEDY BOULEVARD, STE 1800….

[62] Asscher VER, Waars SN, van der Meulen-de Jong AE, Stuyt RJL, Baven-Pronk AMC, van der Marel S, et al. Deficits in geriatric Assessment Associate with Disease Activity and Burden in older patients with inflammatory bowel disease. Clin Gastroenterol Hepatol. 2022;20(5):e1006–21. 34153476 10.1016/j.cgh.2021.06.015

[63] Abautret-Daly Á, Dempsey E, Riestra S, de Francisco-García R, Parra-Blanco A, Rodrigo L, et al. Association between psychological measures with inflammatory anddisease-related markers of inflammatory bowel disease. Int J Psychiatry Clin Pract. 2017;21(3):221–30. 28353360 10.1080/13651501.2017.1306081

[64] Iordache MM, Belu AM, Vlad SE, Aivaz KA, Dumitru A, Tocia C, et al. Calprotectin, Biomarker of Depression in patients with inflammatory bowel disease? Medicina. 2023;59(7):1240. 37512053 10.3390/medicina59071240 PMC10383955

[65] Ghoshal UC, Sahu S, Saigal VM, editors. Patients with ulcerative colitis, particularly during relapse, have more psychological issues and worse quality of life. WILEY 111 RIVER ST, HOBOKEN 07030– 5774, NJ USA: JOURNAL OF GASTROENTEROLOGY AND HEPATOLOGY; 2021.

[66] Geiss T, Schaefert RM, Berens S, Hoffmann P, Gauss A. Risk of depression in patients with inflammatory bowel disease. J Dig Dis. 2018;19(8):456–67. 29989345 10.1111/1751-2980.12644

[67] Eiff B, Bullmore ET, Clatworthy MR, Fryer TD, Pariante CM, Mondelli V, et al. Extra-axial inflammatory signal and its relationship to peripheral and central immunity in depression. Brain. 2025;148(2):635–46. 39657983 10.1093/brain/awae343 PMC11788198

[68] Turkheimer FE, Veronese M, Mondelli V, Cash D, Pariante CM. Sickness behaviour and depression: An updated model of peripheral-central immunity interactions. Brain, Behavior, and Immunity. 2023;111:202– 10. 10.1016/j.bbi.2023.03.031 37076054

[69] Jacobs JP, Sauk JS, Ahdoot AI, Liang F, Katzka W, Ryu HJ, et al. Microbial and metabolite signatures of stress reactivity in ulcerative colitis patients in clinical remission predict clinical flare risk. Inflamm Bowel Dis. 2024;30(3):336–46. 37650887 10.1093/ibd/izad185 PMC10906354

[70] Lee J, Oh SJ, Ha E, Shin GY, Kim HJ, Kim K, et al. Gut microbial and human genetic signatures of inflammatory bowel disease increase risk of comorbid mental disorders. npj Genomic Med. 2024;9(1):52. 10.1038/s41525-024-00440-w PMC11522461 39472439

[71] Liu P, Liu Z, Wang J, Wang J, Gao M, Zhang Y, et al. Immunoregulatory role of the gut microbiota in inflammatory depression. Nat Commun. 2024;15(1):3003. 38589368 10.1038/s41467-024-47273-w PMC11001948

[72] Gao X, Duan S, Cao Y, Zhang Y. Change of monocytes/macrophages in ulcerative colitis patients with symptoms of anxiety and depression. BMC Gastroenterol. 2023;23(1):67. 36906523 10.1186/s12876-023-02693-8 PMC10007821

[73] Tang Y, Zhao L, Lei N, Chen P, Zhang Y. Crohn’s disease patients with depression exhibit alterations in monocyte/macrophage phenotype and increased proinflammatory cytokine production. Dig Dis. 2020;38(3):211–21. 31216541 10.1159/000501122

[74] Dragasevic S, Stankovic B, Kotur N, Sokic Milutinovic A, Nikolic A, Pavlovic S, et al. Psychological distress is associated with inflammatory bowel disease manifestation and mucosal inflammation. Inflammatory Bowel Dis. 2024:izae180. 10.1093/ibd/izae180 39191508

[75] Aniwan S, Park SH, Al-Bawardy B, Kane SV, Coelho-Prabhu N, Kisiel JB, et al. Mo1802-The relationship between patient-reported clinical outcomes, endoscopic inflammation and health-related quality of life in patients with Ulcerative Colitis. Gastroenterology. 2018;6(154):S–806. 7.

[76] Khan M, Calpin P, Kelly O, Moloney J, McDermott E, Mulcahy H, et al. THE EFFECTS OF ENDOSCOPIC SEVERITY AND BIOLOGIC TREATMENT ON LEVELS OF DEPRESSION AND DISABILITY AMONGST IBD PATIENTS: A CASE CONTROL STUDY. Gut. 2013;62(Suppl 2):A43–4.

[77] Lamb CA, Saifuddin A, Powell N, Rieder F. The future of precision medicine to predict outcomes and control tissue remodeling in inflammatory bowel disease. Gastroenterology. 2022;162(5):1525–42. 34995532 10.1053/j.gastro.2021.09.077 PMC8983496

[78] Jagirdhar GSK, Perez JA, Perez AB, Surani S. Integration and implementation of precision medicine in the multifaceted inflammatory bowel disease. World J Gastroenterol. 2023;29(36):5211–25. 37901450 10.3748/wjg.v29.i36.5211 PMC10600960

[79] Han C, Pae CU. Pain and depression: a neurobiological perspective of their relationship. Psychiatry Investig. 2015;12(1):1–8. 25670939 10.4306/pi.2015.12.1.1 PMC4310906

[80] Goesling J, Clauw DJ, Hassett AL. Pain and depression: an integrative review of neurobiological and psychological factors. Curr Psychiatry Rep. 2013;15:1–8. 10.1007/s11920-013-0421-0 24214740

[81] Rochelle TL, Fidler H. The importance of illness perceptions, quality of life and psychological status in patients with ulcerative colitis and Crohn’s disease. J Health Psychol. 2013;18(7):972–83. 23027780 10.1177/1359105312459094

[82] Stapersma L, van den Brink G, van der Ende J, Bodelier AG, van Wering HM, Hurkmans PCWM, et al. Illness perceptions and Depression are Associated with Health-Related Quality of Life in Youth with Inflammatory Bowel Disease. Int J Behav Med. 2019;26(4):415–26. 31183787 10.1007/s12529-019-09791-6 PMC6652166

[83] van der Have M, Fidder HH, Leenders M, Kaptein AA, van der Valk ME, van Bodegraven AA, et al. Self-reported disability in patients with inflammatory bowel disease largely determined by disease activity and illness perceptions. Inflamm Bowel Dis. 2015;21(2):369–77. 25569738 10.1097/MIB.0000000000000278

[84] van der Have M, Minderhoud IM, Kaptein AA, Leenders M, Siersema PD, Fidder HH, et al. Substantial impact of illness perceptions on quality of life in patients with Crohn’s disease. J Crohns Colitis. 2013;7(8):e292–301. 23218732 10.1016/j.crohns.2012.11.002

[85] van Tilburg MAL, Claar RL, Romano JM, Langer SL, Drossman DA, Whitehead WE, et al. Psychological factors may play an important role in Pediatric Crohn’s disease symptoms and disability. J Pediatr. 2017;184:94–e1001. 28238483 10.1016/j.jpeds.2017.01.058 PMC5407185

[86] Crombez G, Van Damme S, Eccleston C. Hypervigilance to pain: an experimental and clinical analysis. Pain. 2005;116(1–2):4–7. 15927387 10.1016/j.pain.2005.03.035

[87] Hughes A, Hirsch C, Chalder T, Moss-Morris R. Attentional and interpretive bias towards illness‐related information in chronic fatigue syndrome: a systematic review. Br J Health Psychol. 2016;21(4):741–63. 27329758 10.1111/bjhp.12207

[88] Kosmidis M. Confirmation bias. Decision Making in Emergency Medicine: Biases, Errors and Solutions. 2021:83– 8.

[89] Quartana PJ, Campbell CM, Edwards RR. Pain catastrophizing: a critical review. Expert Rev Neurother. 2009;9(5):745–58. 19402782 10.1586/ERN.09.34 PMC2696024

[90] Williet N, Sandborn WJ, Peyrin–Biroulet L. Patient-reported outcomes as primary end points in clinical trials of inflammatory bowel disease. Clin Gastroenterol Hepatol. 2014;12(8):1246–56. e6. 24534550 10.1016/j.cgh.2014.02.016

[91] Huisman D, Fernhout F, Moxham F, Norton C, Bannister K, Moss-Morris R. Managing patients’ reports of abdominal pain and irritable bowel syndrome-like symptoms during quiescent inflammatory bowel disease: a role for shared sensemaking. Br J Pain. 2024;18(4):325–336. 10.1177/20494637241230807 PMC11289903 39092211

[92] Wileman V, Chilcot J, Norton C, Hart A, Miller L, Stagg I et al. Modifiable psychological factors are associated with clusters of pain, fatigue, faecal incontinence and IBS-type symptoms in inflammatory bowel disease: a latent profile analysis. J Crohn’s Colitis. 2024;jjae183:1–9. 10.1093/ecco-jcc/jjae183 PMC12087568 39656929

[93] Gandhi S, Jedel S, Hood M, Mutlu E, Swanson G, Keshavarzian A. The relationship between coping, health competence and patient participation among patients with inactive inflammatory bowel disease. J Crohn’s Colitis. 2014;8(5):401–8. 24230968 10.1016/j.crohns.2013.10.005

[94] Cohen J. Statistical power analysis for the behavioral sciences. Academic; 2013.

[95] Beard JA, Click BH. The burden of cost in inflammatory bowel disease: a medical economic perspective. Curr Opin Gastroenterol. 2020;36(4):310–6. 32398566 10.1097/MOG.0000000000000642

[96] Lores T, Goess C, Mikocka-Walus A, Collins KL, Burke ALJ, Chur-Hansen A, et al. Integrated Psychological Care Reduces Health Care Costs at a hospital-based inflammatory bowel Disease Service. Clin Gastroenterol Hepatol. 2021;19(1):96–e1033. 32007538 10.1016/j.cgh.2020.01.030

[97] Birkinshaw H, Friedrich CM, Cole P, Eccleston C, Serfaty M, Stewart G, et al. Antidepressants for pain management in adults with chronic pain: a network meta-analysis. Cochrane Database Syst Rev. 2023;5(5):Cd014682. 37160297 10.1002/14651858.CD014682.pub2 PMC10169288

[98] Bonilla-Jaime H, Sánchez-Salcedo JA, Estevez-Cabrera MM, Molina-Jiménez T, Cortes-Altamirano JL. Alfaro-Rodríguez A. Depression and Pain: Use of antidepressants. Curr Neuropharmacol. 2022;20(2):384–402. 34151765 10.2174/1570159X19666210609161447 PMC9413796

[99] Yasir M, Goyal A, Sonthalia S. Corticosteroid adverse effects. 2018. 30285357

