# Supplementary material for: The relationships between depression, inflammation and self-reported disease activity in IBD and their impact on healthcare usage
Source: BMC Gastroenterol. 2025 Mar 6;25:140. doi: 10.1186/s12876-025-03691-8 (PMC11883936; doi:10.1186/s12876-025-03691-8)
Supplement: Supplementary file 1 — Supplementary Material 1 [file 12876_2025_3691_MOESM1_ESM.docx]

# Supplementary Materials

## Supplementary Material 1. Scatterplot of depression and self-reported disease activity

## Supplementary Material 2. Scatterplot of depression and faecal calprotectin

|  |  |
| --- | --- |

## Supplementary Material 3. Scatterplot of self-reported disease activity and faecal calprotectin

|  |  |
| --- | --- |

## Supplementary Material 4. Results of regression models for health service (n=599), sick leave days (n=327) and impact of IBD on productivity at work (n=336) with depression, inflammation and self-reported disease control entered as predictor variables.

|  | Model significance | Adjusted r Square | Depression (PHQ-9)  β (95%CI) | Faecal calprotectin  β (95%CI) | SRDA (IBD-Control)  β (95%CI) | Crohn’s Disease  β (95%CI) | Diagnosis X FCP  β (95%CI) |
| --- | --- | --- | --- | --- | --- | --- | --- |
| Primary care visits | F(5,593) = 9.74 | .068*** | .189 (.091, .287)*** | .109 (-.005, .223) | -.093 (-.090, .050) | .237 (.454, .020)* | -.246 (-.478, -.013)* |
| Secondary care visits | F(5,593) = 18.85 | .130*** | .130(.036, .224)** | .265 (.155, .376)*** | -.196 (-.292, -.100)*** | .202 (-.007, 4.12) | -.271 (-.495, -.047)* |
| A&E visits | F(5,593) = 3.37 | .019** | .101 (.001,.201)* | -.000 (-.117, .117) | -.054 (-.155, .048) | -.111 (-.334, .111) | .157 (-.081, .395) |
| Total visits | F(5,593) = 21.64 | .147*** | .179 (.086, .273)*** | .250 (.141, .359)*** | -.191 (-.286, -.097)*** | .242 (.035, .450)* | -.297 (-.519, -.075)** |
| Sick leave days ^Ѱ^ | F(5,331) = 2.87 | .028* | .055 (-.089, .200) | .002 (-.157, .151) | -.149 (-.287, -.012)* | .015 (-.279, .309) | -.076 (-.235, .386) |
| Impact on productivity ^Ѱ^ | F(5,330) = 31.56 | 0.313*** | .235 (.117, .354)*** | -.028 (-.101, .157) | -0.415 (-.528, -.301)*** | -.048 (-.292, .197) | .028 (-.230, .286) |

*** indicates p<0.001, ** p<0.01, * p<0.05

^Ѱ^ analysis only conducted in proportion of sample in employment (i.e., not retired or not working)

A&E=accident and emergency, FCP=faecal calprotectin, IBD=inflammatory bowel disease, PHQ-9=Patient Health Questionnaire-9, SRDA=self-reported disease activity.
